# Lesion size and circumferential range identified as independent risk factors for esophageal stricture after endoscopic submucosal dissection

**DOI:** 10.1007/s00464-020-07368-z

**Published:** 2020-01-17

**Authors:** Meihong Chen, Yini Dang, Chao Ding, Jiajia Yang, Xinmin Si, Guoxin Zhang

**Affiliations:** Department of Gastroenterology, Jiangsu Province Hospital and The First Affiliated Hospital of Nanjing Medical University, First Clinical Medical College of Nanjing Medical University, 300 Guangzhou road, Gulou district, Nanjing, Jiangsu China

**Keywords:** Endoscopic submucosal dissection (ESD), Esophageal stenosis, Risk factors, Retrospective

## Abstract

**Background and aim:**

Endoscopic submucosal dissection (ESD) is used to treat early esophageal cancer and precancerous lesions. Patients undergoing ESD are prone to esophageal stenosis, which impairs therapeutic efficacy and quality of life. This retrospective study aimed to investigate the potential association between patient demographics and esophageal lesion characteristics with the risk of esophageal stenosis following ESD.

**Methods:**

For this retrospective study 190 consecutive patients who underwent ESD between January 2013 and January 2015 were recruited. Data on patient demographics, esophageal lesion-related factors, operation details, esophageal stenosis occurrence and measures taken to prevent or treat stricture were collected, and the normality of distribution of each indicator was assessed with a Kolmogorov–Smirnov test. Stenosis risk factors were then identified using univariate and multivariate logistic regression.

**Results:**

Post-ESD esophageal stenosis occurred in 51 cases. Multivariate logistic regression analysis was performed to identify independent risk factors. A history of EMR/ESD (OR = 4.185, 95% CI: 1.511–11.589), resection circumferential diameter (OR = 1.721, 95% CI: 1.135–2.610), non-*en bloc* resection (OR = 7.413, 95% CI: 2.398–22.921), submucosal infiltration (OR = 3.449, 95% CI: 1.014–11.734) and circumferential resection range (OR = 57.493, 95% CI: 17.236–191.782) were identified as independent risk factors for post-ESD esophageal stenosis. Spraying porcine fibrin adhesive on the resection bed reduced neither the incidence of postoperative stenosis nor the extent of postoperative dilation.

**Conclusion:**

Post-ESD esophageal stenosis is significantly related to size and circumferential range of lesion resection. EMR/ESD history, non-*en bloc* resection and submucosal infiltration may be additional risk factors.

Early esophageal cancer and precancerous lesions are confined to the submucosa, with low risk of distant metastasis. Endoscopic resection has become the first-line therapy for the treatment of early esophageal neoplasia. Unlike endoscopic mucosal resection (EMR), endoscopic submucosal dissection (ESD) allows *en bloc* resection regardless of lesion size, reducing the risk of recurrence and facilitating precise histologic staging. Therefore, ESD may be superior to EMR in the treatment of early esophageal cancer, due to significantly higher *en bloc* and curative resection rates and lower local recurrence rates [1, 2]. Furthermore, ESD can effectively remove lesions, leading to less trauma, fewer complications, no reported mortality, and higher quality of life for patients. The long-term efficacy of ESD is comparable to that of surgery, so ESD has become the standard therapeutic technique for early esophageal cancer [3, 4]. However, it is noteworthy that acute inflammatory reaction and fibrous connective tissue hyperplasia following extensive endoscopic resection can lead to esophageal stenosis, changes in eating behavior, and aspiration pneumonia in patients [5]. There is currently no consensus regarding the relationship between ESD and the development of postoperative esophageal stricture.

## Methods

### Patient selection

Relevant information on patients undergoing esophageal ESD between January 2013 and January 2015 was provided by the electronic medical records of the Jiangsu Provincial People's Hospital. A total of 229 patients underwent esophageal ESD, regardless of the pre- or postoperative pathology. In China, the incidence of esophageal squamous cell carcinoma (including high/low grade intraepithelial neoplasia) is considerably higher than that of adenocarcinoma and Barrett's esophagus. In Europe and the United States, the predominant histologic subtype is adenocarcinoma [6]. In order to reduce heterogeneity of the patient population, patients with Barrett's esophagus or adenocarcinoma were excluded. The inclusion criteria for the study were as follows: (1) The pathological diagnosis was low grade intraepithelial neoplasia (LGIN), high grade intraepithelial neoplasia (HGIN), or squamous cell carcinoma; (2) No additional surgery or chemoradiotherapy was performed after ESD.

This study has been approved by the Ethics Committee of the Jiangsu Provincial People's Hospital and written informed consent was obtained from each patient. The ethical approval number for this study was 2018-SR-213.

### Data collection

All patients had a follow-up exam within six months of undergoing ESD. The outcome of interest at follow-up was the presence of esophageal stenosis. The enrolled patients were divided into stenosis and non-stenosis groups based on the following definition of esophageal stenosis: narrowing of the esophageal lumen to the extent that it becomes impassable by an ordinary endoscope (diameter 9.8 mm) and an accompanying difficulty in consuming solid foods [7]. Demographic information including age, sex, body mass index (BMI), smoking history, drinking history, comorbidity and family history of esophageal cancer was collected. Lesion characteristics including pre- and postoperative pathology, location, lesion number, ultrasonic infiltration, morphology, longitudinal resection length, circumferential specimen size, circumferential range and depth of infiltration were recorded. Procedural details including ‘lifting sign’, electric coagulation, *en bloc* resection, muscular injury, perforation, hemorrhage, clip number, and operating time were also collected.

### Statistical analysis

Statistical analysis was performed using the SPSS Statistics 20.0 program (SPSS, Chicago, Illinois, USA). Due to the large number of potential risk factors, we first screened the variables with univariate analysis and identified those that appeared to differ between the stenosis and non-stenosis groups (*P* < 0.05). Firstly, we used the Kolmogorov–Smirnov test to analyze the normality of the distribution of each indicator. For normally distributed continuous variables, a Student’s *t*-test was then conducted to assess the intergroup differences and results were reported as mean ± SD. The Wilcoxon rank-sum test was used for continuous non-normal data. For discrete data, the Fisher’s exact and chi-squared tests were performed in order to analyze differences between proportions in the stenosis and non-stenosis groups.

We then conducted multivariate logistic regression analyses with the significant variables in order to evaluate the association between esophageal stricture and potential risk factors.

## Results

A total of 190 patients, with 222 lesions, fulfilled the inclusion criteria and entered the analyses. There were 51 patients (26.8%) who developed stenosis by the time of follow-up, whereas no stenosis was present in 139 patients (73.2%) at follow-up. Patients were divided into two groups for analyses, namely a stenosis group and a non-stenosis group. Demographic information, lesion characteristics and procedural details for each of the two groups are depicted in Tables 1–5.

**Table 1:** Univariate analysis of patient characteristics

Patient characteristics	Non-stenosis	Stenosis	*P* value
Number, *n*	139	51	
Lesion number, *n*	160	62	0.889
Age, mean ± SD, year	63.4 ± 7.5	64.8 ± 8.1	0.278
Sex, male/female, *n*	100/39	32/19	0.222
BMI, mean ± SD	23.2 ± 3.1	23.3 ± 2.9	0.882
Smoking history, *n*	54	13	0.088
Drinking history, *n*	42	14	0.711
Comorbidity, *n*			
Hypertension	38	17	0.419
Diabetes	8	1	0.48
Coronary heart disease	5	2	1
Stroke	5	5	0.183
COPD	4	1	1
Gallbladder surgery	9	0	0.14
Gastrointestinal EMR/ESD	17	15	0.005*
Other cancers	11	4	1
Family history, *n*	22	8	0.981

*BMI* Body Mass Index, *COPD* chronic obstructive pulmonary disease

*Indicates that the results were statistically significant

### Univariate analysis of demographic information

The mean age for the non-stenosis group was similar to that of the stenosis group (63.4 ± 7.5 years vs. 64.8 ± 8.1 years, *P* = 0.278). The number of male patients was twice the number of females in each group. Univariate analyses highlighted that a history of gastrointestinal EMR/ESD (17 vs. 15, *P* = 0.005) was significantly different between the two groups; patients in the non-stenosis group were less likely to have a history of gastrointestinal EMR/ESD compared to those in the stenosis group. However, there were no significant differences found between the two groups in lesion number, age, sex, BMI, smoking history, drinking history, hypertension, diabetes, coronary heart disease, stroke, chronic obstructive pulmonary disease (COPD), gallbladder surgery, other cancers or family history (Table 1).

### Univariate analysis of lesion characteristics

There were no significant differences between the two groups regarding the lesion location, lesion number, degree of ultrasonic infiltration, or morphology (*P* > 0.05). However, there were significant differences found between the two groups regarding preoperative pathology, longitudinal resection length, circumferential specimen size, circumferential range, postoperative pathology and depth of infiltration (*P* < 0.05) (Table 2). We performed further subgroup analysis based on circumferential range, as preoperative pathology had not been mentioned in previous studies as a possible risk factor for the development of esophageal stenosis. However, we did not identify any conspicuous differences in preoperative pathology between each subgroup (Table 3).

**Table 2:** Univariate analysis of lesion characteristics

Lesion characteristics	Non-stenosis	Stenosis	*P* value
Preoperative pathology, *n*			0.001*
LGIN	56	8	
HGIN	83	43	
Location, *n*			0.698
Upper thoracic part	6	3	
Middle thoracic part	78	31	
Lower thoracic part	55	17	
Lesion number, *n*			0.103
Single	120	39	
Multiple	19	12	
Ultrasonic infiltration, *n*			0.357
Mucosal	97	32	
Submucosal	42	19	
Morphology			0.774
Flat	129	46	
Protruded	10	5	
Depressed	0	0	
Longitudinal resection length, medium (cm)	6	7	< 0.001*
Circumferential specimen size, medium, (cm)	3	4	< 0.001*
Circumferential range, *n*			< 0.001*
< 1/2	71	1	
1/2–3/4	65	9	
3/4–1	3	41	
Postoperative pathology, *n*			< 0.001*
LGIN	30	0	
HGIN	94	36	
Tis	14	10	
Squamous carcinoma	1	5	
Depth of infiltration, *n*			< 0.001*
Mucosal	131	39	
Submucosal	8	12	

*LGIN* low grade intraepithelial neoplasia, *HGIN* high grade intraepithelial neoplasia, *Tis* refers to carcinoma in situ

*Indicates that the results were statistically significant

**Table 3:** Subgroup analysis of preoperative pathology

	Non-stenosis	Stenosis	*P* value
< 1/2			
Preoperative pathology, *n*			0.408
LGIN	29	0	
HGIN	42	1	
1/2–3/4			
Preoperative pathology, *n*			0.701
LGIN	26	3	
HGIN	39	6	
3/4–1			
Preoperative pathology, *n*			0.303
LGIN	1	5	
HGIN	2	36	

*LGIN* low grade intraepithelial neoplasia, *HGIN* high grade intraepithelial neoplasia

### Univariate analysis of factors related to ESD procedure

Specific factors related to the ESD procedure may influence the risk of developing postoperative esophageal stenosis. Univariate analysis of procedure characteristics indicated that lift sign, repeated electric coagulation, *en bloc* resection, muscular injury, perforation and operation time were associated with the presence of stenosis at follow-up (*P* < 0.05), whereas hemorrhage and number of clips were not (*P* > 0.05) (Table 4).

**Table 4:** Univariate analysis of procedure characteristics

Procedure characteristics	Non-stenosis	Stenosis	*P* value
Lift sign*, n*			0.017*
Positive	136	45	
Negative	3	6	
Rich blood vessels, *n*			0.033*
Rich	106	46	
Not rich	33	5	
*En bloc* resection, *n*			< 0.001*
Yes	130	32	
No	9	19	
Muscular injury*, n*			0.005*
Yes	21	17	
No	118	34	
Perforation, *n*			< 0.001*
Yes	0	6	
No	139	45	
Hemorrhage*, n*			0.104
Yes	1	3	
No	128	48	
Clips number	2	3	0.81
Operating time, medium, min	60	90	< 0.001*
Experience of operator, *n*			0.971
Average	13	4	
Advanced	126	47	

*Indicates that the results were statistically significant

Results from the univariate analyses therefore suggest that the following factors are associated with the development of postoperative esophageal ESD stenosis: a history of gastrointestinal EMR/ESD, preoperative pathology, longitudinal resection length, maximum specimen size, circumferential range, postoperative pathology, depth of infiltration, lift sign, repeated electric coagulation, *en bloc* resection, muscular injury, perforation and operation time.

### Multivariate logistic regression analyses

The above indicators that were identified as being significantly different between the stenosis and non-stenosis group through univariate analyses were then included in multivariate analyses for further verification. Logistic regression analyses determined that a history of gastrointestinal EMR/ESD (OR = 4.185, 95% CI: 1.511–11.589), circumferential specimen size (OR = 1.721, 95% CI: 1.135–2.610), circumferential range (OR = 57.493, 95% CI: 17.236–191.782), depth of infiltration (OR = 3.449, 95% CI: 1.014–11.734) and non-*en bloc* resection (OR = 7.413, 95% CI: 2.398–22.921) were independent risk factors for postoperative stenosis at follow-up (Table 5).

**Table 5:** Multivariate analysis

Risk factors	OR	95% CI	*P* value
Pre-ESD history	4.185	1.511–11.589	0.006
Maximum specimen size	1.721	1.135–2.610	0.011
Circumferential range	57.493	17.236–191.782	< 0.001
Depth of infiltration	3.449	1.014–11.734	0.048
Non-*en bloc* resection	7.413	2.398–22.921	0.001

*OR* odds ratio, *CI* confidence interval

### Prevention and treatment of esophageal stricture

All patients admitted to the study were routinely treated with proton pump inhibitors (PPI) therapy and mucosal protectants after ESD. In addition, a total of 34 patients in our retrospective study were treated with a porcine fibrin adhesive during ESD. Eleven of them developed esophageal stenosis. It seemed that using porcine fibrin adhesive was not associated with lower incidence of stenosis (OR = 1.387, 95% CI: 0.621–3.097), nor the mean number of stenosis treatments (3.1 ± 4.2 vs 4.2 ± 5.3, *P* = 0.480). No patients included in this study received intraoperative prophylactic local steroid injections. Patients who took oral steroids were not found to have a lower incidence of stenosis, or lower number of dilations, compared to those who did not take steroids (3.1 ± 4.2 vs 7 ± 7.9, *P* = 0.142), so it does not make sense now.

A total of 43 patients in the stenosis group received post-stenosis treatment. The treatment methods included Bougie expansion, stent implantation, expansion combined with stent implantation and expansion combined with drug injection. We found that different treatments were associated with different outcomes, but we could not determine which was the best treatment (Table 6).

**Table 6:** Treatment received for esophageal stenosis

Measures	*N*	The number of treatments	*P* value
Expansion	21	2.1 ± 1.5	
Stent implantation	2	1 ± 0.01	
Expansion + stent	17	6.5 ± 6.3	
Expansion + injection	3	4.3 ± 2.3	
Total	43	4.0 ± 4.6	0.02*

*N* the number of patients

*Indicates that the results were statistically significant

## Discussion

Although ESD has become the leading treatment for early esophageal lesions, the occurrence of postoperative stricture has a significant influence on overall prognosis [8]. Generally, esophageal stricture occurs within 3 weeks after ESD, and the 6-month follow-up is sufficient for the observation of patients. The stenosis rate of this retrospective study was 26.8% (51/190), as shown in Table 7; our classification of stenosis was based on the definition used in relevant literature [7]. A total of 8 patients in the stenosis group did not receive stenosis treatment after surgery, and were found to have mild esophageal stenosis at follow-up. If we exclude the eight untreated patients, the incidence of stenosis in this retrospective study is only 22.6%. Factors contributing to the relatively high rate of stenosis in our retrospective study may include the relatively small sample size compared to that of other studies [9] and the presence of particularly large lesions in 44 patients in our research. Previous literature suggests that the incidence of stenosis is between 80-100% in patients with a circumferential mucosal defect of more than three-quarters [8–15]. Therefore, it is plausible that our study found a higher incidence of total esophageal stricture due to the high percentage of patients presenting with wide esophageal lesions.

**Table 7:** Rate of esophageal stenosis for the retrospective study

	Cases of stenosis	Cases of non-stenosis	Prevalence of stenosis (%)
< 3/4	10	136	6.8
> 3/4	41	3	93.2
Total	51	139	26.8

Overall, the results of this retrospective study indicate a number of independent risk factors for the development of esophageal stenosis following ESD, which are in line with findings of other studies [13–21]. Mizuta et al. [13] highlighted that the primary contributor to esophageal stenosis following ESD was the area of periesophageal mucosal defect exceeding 71%. This study also suggested that circumferential range (OR = 57.493, 95% CI: 17.236–191.782) and mucosal defect circumference length (OR = 1.721, 95% CI: 1.135–2.610) are independent risk factors for esophageal stricture. We found that the incidence of stenosis was 70.1% in patients with mucosal defect of more than 2/3 of the circumferential range. Our retrospective study also suggests that the depth of infiltration (OR = 3.449, 95% CI: 1.014–11.734) might be a reliable independent predictor of postoperative stricture, which is consistent with the conclusions of Ono et al*.* [15].

This study also suggests that patients with a history of gastrointestinal ESD or EMR (OR = 4.185, 95% CI: 1.511–11.589) were more likely to have developed stenosis at follow-up; however, the mechanism underlying this association is unclear and requires further exploration. It is worth noting that we did not conduct an in-depth analysis of patients with a history of ESD treatment; further analyses should include the stratification of patients according to whether they underwent an esophageal, gastric, or intestinal ESD procedure. Additionally, non-*en bloc* resection (OR = 7.413, 95% CI: 2.398–22.921) was considered an independent risk factor. Segmental excision has many potential risks; for example, the risk of tumor recurrence is much higher with fractional resection than with *en bloc* resection [1, 2]. It is difficult to manage resection targets endoscopically and achieve precise resection of tumors and mucosa [22, 23]. Therefore, we recommend that endoscopists aim to achieve *en bloc* resection when performing ESD.

Current recommendations suggest that esophageal stenosis is treated with conventional therapies such as endoscopic balloon dilation [24], stents [25] and glucocorticoid administration [26]. Novel strategies such as stem cell therapy [27], autologous epithelial cell membrane repair [28] and gastric mucosal transplantation [29] have recently emerged; however, none of these methods can completely prevent or alleviate esophageal stenosis. This current study suggests that the main function of porcine fibrin adhesive is for hemostasis, wound sealing, the promotion of healing and the prevention of adhesion during ESD, rather than for preventing stenosis. The preventive effect of oral steroids suggested in other studies [30, 31] was not apparent in our study. In addition, our results are statistically limited due to the inconsistent number of patients in each treatment group. Due to the uneven distribution of study population among groups, and the limited data available, it is difficult to make pairwise comparisons. It is therefore not possible to identify the most effective postoperative treatment.

A history of gastrointestinal ESD should be considered a risk factor for the development of esophageal stenosis, and endoscopists should aim to achieve *en bloc* resection when possible. We suggest a re-evaluation of the indications for esophageal ESD, especially with regards to lesions with wide range and depth. Moreover, our study highlights the importance of a high level of expertise amongst endoscopic surgeons carrying out ESD, to ensure precision, accuracy in locating lesions, and the complete resection of lesions. In a nutshell, this study identified relative risk factors contributing to stenosis formation, and provides suggestions for improving postoperative recovery. In the future, it will provide a theoretical foundation for the prevention and treatment of postoperative esophageal stenosis.

### Strengths and limitations

This retrospective study takes a wide range of indicators, regarding the general condition of the patient, lesion characteristics and ESD operational details, into consideration in analyses in order to comprehensively assess a wide range of possible risk factors associated with postoperative stricture formation. It builds upon previous studies by taking more potential risk factors into consideration. It also considers several risk factors that have not been previously explored in analyses, such as the possible association between a history of gastrointestinal EMR/ESD and stenosis following esophageal ESD. Additionally, we compared the methods of prevention and treatment of stenosis for patients in this study.

Retrospective studies are susceptible to selection bias and recall bias, and the definition of symptoms or diseases may change over time, as was the case in this study with the definition of esophageal stenosis. Due to limited data, only preliminary information is obtained regarding potential risk factors affecting the formation of stenosis, and analyses do not include risk factor stratification.

We conclude that the development of esophageal stenosis following ESD is associated with lesion depth and extent.
